# Effect of 8-week Shoulder External Rotation Exercise with Low Intensity and Slow Movement on Infraspinatus

**DOI:** 10.1298/ptr.E10227

**Published:** 2023-04-22

**Authors:** Aoi MATSUMURA, Hiroshige TATEUCHI, Masatoshi NAKAMURA, Noriaki ICHIHASHI

**Affiliations:** ^1^TAKE PHYSICAL CONDITIONING Inc., Japan; ^2^Human Health Sciences, Graduate School of Medicine, Kyoto University, Japan; ^3^Faculty of Rehabilitation Sciences, Nishi Kyushu University, Japan

**Keywords:** Rotator cuff, Infraspinatus, Low intensity, Slow movement, Hypertrophy

## Abstract

Objective: Generally, low-intensity training is recommended as selective training of the infraspinatus muscle. This study aimed to investigate whether an 8-week intervention of low-intensity, slow-movement, external rotation exercise of the shoulder led to an increase in muscle strength with shoulder external rotation and cross-sectional area (CSA) infraspinatus muscle. Methods: Sixteen healthy male volunteers were randomly assigned to the low-intensity and slow-movement (LS) group (N = 8) or the normal-intensity and normal-speed (NN) group (N = 8). The LS and NN groups performed shoulder external rotation exercises with low intensity and slow movement, and normal intensity and normal speed, respectively. The exercise session consisted of three sets of 10 repetitions, which were performed three times per week for 8 weeks. We measured the CSA of the infraspinatus and muscle strength of the shoulder external rotation before and after the 8-week intervention. Results: A significant increase in infraspinatus CSA from baseline to 8 weeks was found in the LS group (7.3% of baseline) but not in the NN group. No significant differences were found in the muscle strength of shoulder external rotation. Conclusion: Our results suggest that low-intensity exercise of the infraspinatus is effective for muscle hypertrophy when performed with slow movement. This finding may help patients who should avoid excessive stress in the early phase of rehabilitation.

**T**wo stability mechanisms in shoulder function are identified: static and dynamic. Static stabilizers consist of anatomical structures such as the capsules, ligaments, and labrums, whereas dynamic stabilizers consist of rotator cuff and periscapular muscles^1^^,^^2)^. Given their functions as dynamic stabilizers, the coordinated movement of the deltoid and rotator cuff muscles is essential for smooth shoulder joint movement. Specifically, the infraspinatus, subscapularis, and teres minor muscles stabilize the humeral head against the glenoid and provide a fulcrum for the actions of the deltoid and supraspinatus muscles^3)^. In the transverse plane, the balance of the infraspinatus and subscapularis controls the anteroposterior movement of the humeral head^4)^. Infraspinatus dysfunction is known to decrease the stability of the humeral head, causing superior migration and impingement of the subacromial structures. A previous study reported that a patient with subacromial impingement also had infraspinatus dysfunction^5)^.

Previous studies have investigated the activity of the infraspinatus during shoulder exercises using electromyography (EMG)^6^^–^^9)^. Bitter et al. reported that low-intensity shoulder external rotation was appropriate to optimize the relative contribution of the infraspinatus while minimizing deltoid activity^7)^. Moreover, several studies have investigated the effect of low-intensity exercise on the infraspinatus (full can, empty can, shoulder external rotation, etc., with an elastic band or lightweight dumbbell) and found no significant and/or a very small increase in shoulder external rotator muscle strength^10^^–^^12)^. However, none of these studies have investigated the effect of low-intensity exercise on infraspinatus cross-sectional area (CSA). Therefore, although low-intensity training is frequently recommended for selective infraspinatus training, the effect of this training on increasing infraspinatus muscle strength and CSA remains unclear.

Recent studies have suggested that low-intensity resistance exercises with slow movement and tonic force generation can increase muscle strength and CSA, which have mainly been investigated in the lower extremity^13^^–^^15)^. This low-intensity exercise is a safe training method because of less stress on the joints^14)^. Low-intensity exercises are recommended for selective infraspinatus activation and avoidance of overloading the glenohumeral joint; therefore, we hypothesized that low-intensity exercises performed with slow movement can increase muscle strength and CSA of the infraspinatus.

This study aimed to investigate whether 8 weeks of low-intensity shoulder external rotation exercises, performed with slow movement, would lead to an increase in the CSA and muscle strength of the infraspinatus. Previously, we have shown that low-intensity and slow-movement external rotation exercise caused greater stress on the infraspinatus compared to the stress caused by normal-intensity and normal-speed condition^16)^. In addition, low-intensity and normal-speed condition did not cause the stress on infraspinatus. Therefore, we selected low-intensity and slow-movement condition and normal-intensity and normal-speed condition, which are expected to have training effects on the infraspinatus in this study.

## Methods

### Study design

**Fig. 1.**
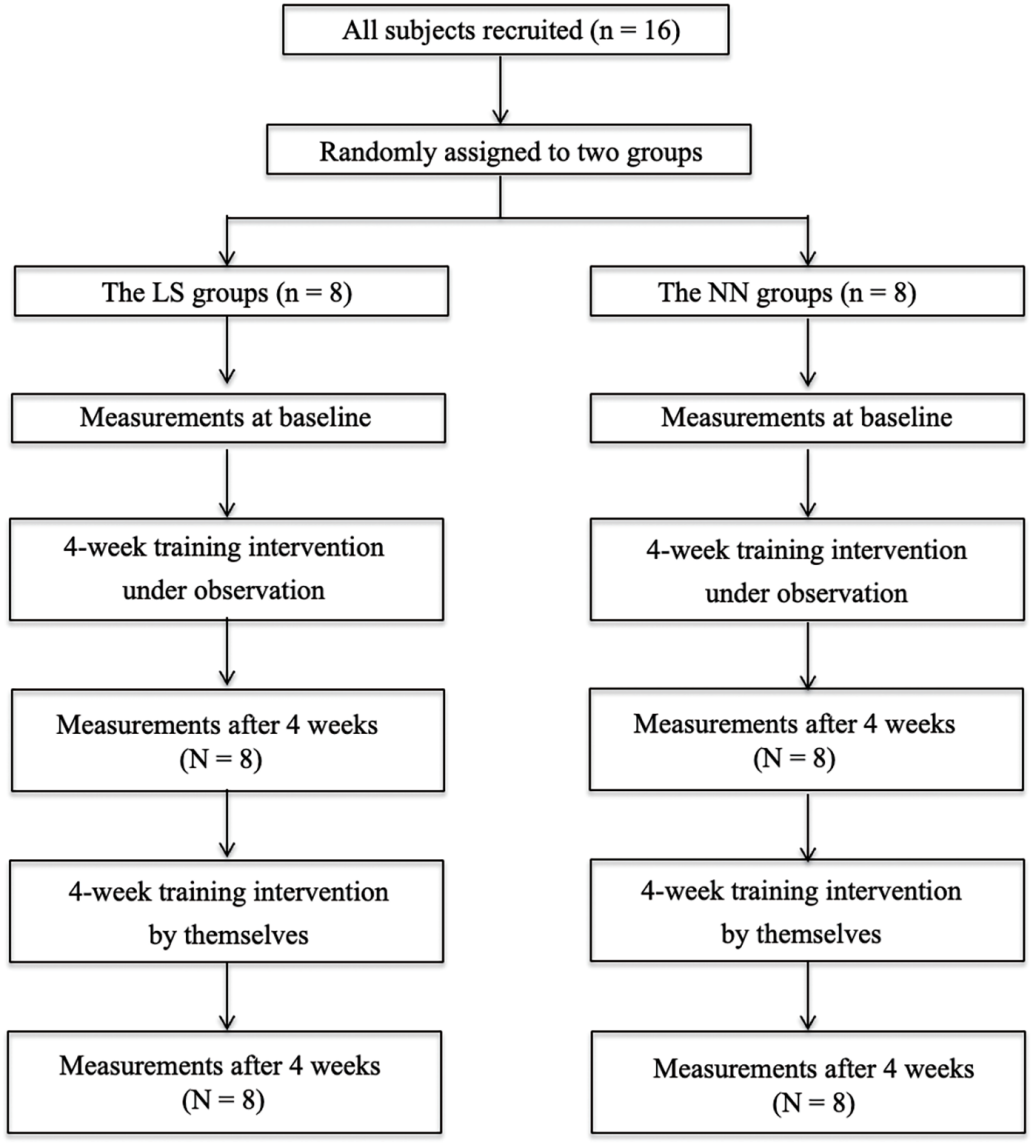
Flow chart of the experimental protocol

LS, low intensity and slow movement; NN, normal intensity and normal speed

This randomized controlled study examined the effects of low-intensity exercises with slow movement performed for 8 weeks and was conducted in accordance with the CONSORT (Consolidated Standards of Reporting Trial) statement^17)^. Figure 1 shows the experimental protocol.

As baseline evaluation, we measured infraspinatus CSA using ultrasonography, isometric shoulder external rotation strength using a handheld dynamometer, and isokinetic external rotation strength using a dynamometer. Then, the participants were randomly assigned to one of the two experimental groups. The exercise session consisted of three sets of 10 repetitions, which were performed three times per week for 8 weeks. Researchers supervised the participants for the initial 4 weeks. After 4 weeks of evaluation, the participants performed the exercises on their own. The infraspinatus CSA and muscle strength of the shoulder external rotation were measured after 4 and 8 weeks of exercise. To avoid the acute effects of exercise, all measurements were performed at least 48 h after the most recent exercise session. Additionally, an EMG surface electrode was used to record the activities of the infraspinatus and posterior deltoid muscles in the first exercise session.

### Participants

Sixteen healthy male volunteers, aged 22–32 years, participated in this study and were not involved in any upper extremity strength training or any kind of regular overhead sports during the study. Participants with a history of musculoskeletal injury or neuromuscular disease involving their upper arms were excluded. A power of 0.80, alpha level of 0.05, effect size F of 0.4 (large), and number of measurements of 3 were assumed for the analysis of variance for the split-plot factorial design, which determined the sample size of 12 in total. All participants were fully informed of the procedures and purpose of this study, and they provided written informed consent. Additionally, the Ethical Review Board of Kyoto University Graduate School of Medicine approved this research (E1407).

### Procedures

#### Measurement of infraspinatus CSA

**Fig. 2.**
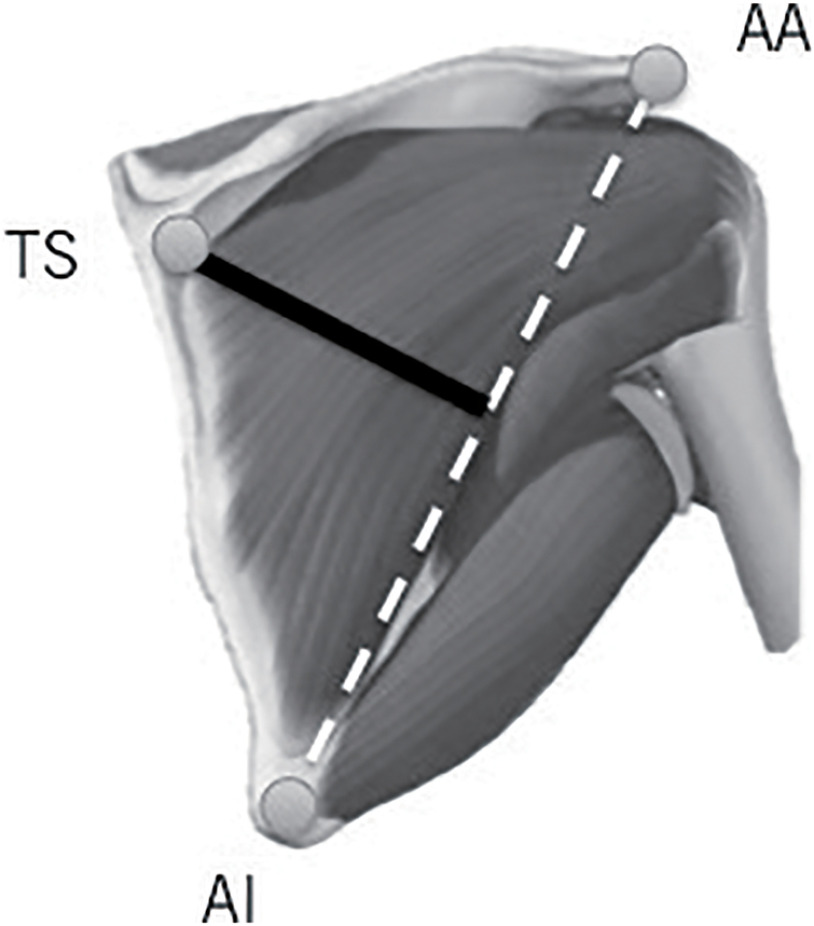
Measuring place of infraspinatus CSA

TS, trignum spinae; AA, acromial angle; AI, inferior angle; CSA, cross-sectional area

**Fig. 3.**
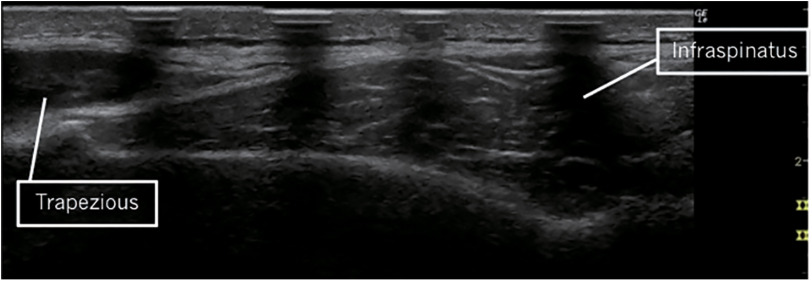
Ultrasound image of the infraspinatus muscle constructed from the series of overlapping images

B-mode ultrasonography (LOGIQ Book XP; GE Healthcare Japan, Tokyo, Japan) with an 8-MHz linear probe was used to obtain images of the infraspinatus and measure CSA. The infraspinatus CSA was measured as described in a previous study^18)^. Briefly, the participants lay prone on the treatment table, with their arms on their sides. To determine the standardized location for the ultrasound, the acromial angle, trigonum spinae, and inferior angle of the scapula were identified through palpation and marked with a marker. After the landmarks were identified, an investigator drew a line connecting the acromial and inferior angles and a second line perpendicular to the first line, intersecting the trigonum spinae (Fig. 2). Hypoechoic markers were placed along the second line. We constructed the full image of the infraspinatus from a series of overlapping images guided by the hypoechoic markers using Adobe Photoshop (Adobe, San Jose, CA, USA) (Fig. 3). Then, the CSA was measured by tracing the inside of the epimysium of the infraspinatus using ImageJ software (National Institutes of Health, Bethesda, MD, USA)^18)^. The average of three measurements of the infraspinatus CSA was calculated to express the values for each participant in each session, in which the experimenter was blinded for the group allocation and timing of measurement. We also calculated the percentage of change in the infraspinatus CSA using the following equation:

Percentage of change (%) = (value at 8 weeks − value at baseline)/value at baseline × 100.

#### Measurement of muscle strength

Isometric and isokinetic strength of the shoulder external rotator muscles was assessed. The maximal isometric shoulder external rotation torque was measured using a handheld dynamometer (PowerTrack II MMT Commander; JTECH Medical, Midvale, USA). The participants sat on a chair, with their back upright, arms by their side in shoulder neutral rotation position, and elbows flexed to 90. The dynamometer was placed distal to the radius. Each subject made two times of maximal efforts during muscle strength tests, and we used the greater value for analysis. Strong verbal encouragement was provided during every contraction to promote maximal effort.

The maximal isokinetic shoulder external rotation torque was measured using a dynamometer (MYORET RZ-450; Kawasaki Heavy Industries, Kobe, Japan). The participants lay in the supine position with the shoulder abducted 90° and the elbow flexed 90°. The shoulder joint was aligned with the axis of rotation of the dynamometer shaft, and the external rotation ranged 0°–90°. The torso and upper arms were fastened with a belt to avoid shoulder flexion or abduction. Then, both the concentric and eccentric maximal torques in shoulder external rotation were evaluated. After five practice repetitions, a test of two consecutive maximal efforts was conducted at 60°/s. After adequate rest, participants performed two consecutive maximal efforts at 120°/s. We used the greater peak torque in the test session for the analysis.

### EMG

During the first session of exercise, an EMG (TeleMyo 2400; Noraxon USA, Scottsdale, AZ, USA) surface electrode (BlueSensor M; Ambu, Copenhagen, Denmark) was used to record muscle activity from the infraspinatus and posterior deltoid. Before the surface electrodes were applied, the participant’s skin was prepared to reduce skin impedance. The surface electrode for the infraspinatus was positioned at the point halfway along the scapular spine and halfway down toward the inferior apex of the scapula, parallel to the muscle fibers^19)^. Maximal voluntary isometric contractions (MVCs) were performed for 3 s to normalize EMG data, as previously described for manual muscle testing^7)^. We provided strong verbal encouragement during every contraction to promote maximal effort.

The EMG data were sampled at 1500 Hz. Raw EMG signals were digitally filtered (band-pass filtered at 20–500 Hz) and were smoothed using a moving root mean square with a window of 50 ms. These EMG data were normalized and expressed as a percentage of their MVC. EMG values for each muscle were averaged across the eight intermediate repetitions of the 10 repetitions completed (% MVC). We also calculated an integrated EMG value for each muscle in the set of exercises and calculated the average of the three sets in the first exercise session (% MVC·s).

### Exercise task

Previous studies examining the effects of low-intensity with slow-movement exercise in lower extremity muscles used 50% of the 1 repetition maximum (RM) as the low intensity. To prevent overstressing the joints, low-intensity exercise is generally considered more appropriate for rotator cuff muscles in rehabilitation^20^^,^^21)^. In previous studies examining the effects of low load exercise for rotator cuff muscles, elastic band and 3-kg dumbbell were used^10^^–^^12)^. However, even these loadings are considered too high for patients with shoulder disorders in the early phase of rehabilitation. Therefore, we used a 500-g dumbbell as low intensity in this study.

**Fig. 4.**
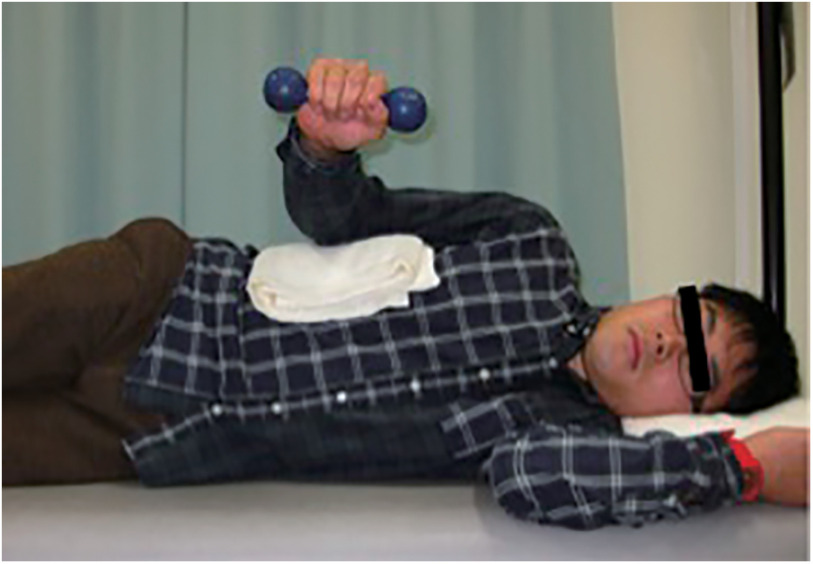
Exercise task

Between the two experimental groups, one group (low intensity and slow movement: LS) exercised with low intensity (dumbbell of 500 g) and slow movement (5 s of external rotation, 5 s of internal rotation, and 1 s of isometric actions with no rest between each repetition). The other group (normal intensity and normal speed: NN) exercised with normal intensity (dumbbell of 2.5 kg) and normal speed (1 s of external rotation and 1 s of internal rotation with 1 s of rest on a table between each repetition). The exercises targeted shoulder external rotation and were performed with the arm by the side and the elbow flexed 90° while lying on the side (Fig. 4). Participants held a towel under their arms to avoid trick motion. The range of shoulder joint motion for shoulder external rotation exercise was set from 45° internal rotation to 45° external rotation. Both groups repeated the movements at approximately constant speed and frequency by following a metronome.

### Statistical analysis

All statistical analyses were performed with SPSS Statistics version 17.0 (SPSS, Chicago, USA). Differences between the LS and NN groups, including participant characteristics and all variables at baseline, were assessed using an independent t-test. For the measurements of the CSA and muscle strength, split-plot analyses of variance using two factors (groups × test time) were used to analyze the interaction and main effects. When we obtained a significant interaction, we used a paired t-test with the Holm method correction to determine the differences between the variables at baseline and after 4 and 8 weeks within the groups. Additionally, we used the Mann–Whitney U test to compare EMG values. Significance was accepted at p <0.05.

## Results

Sixteen participants were recruited from July 30 to September 20, 2012. No participants were excluded from the study, and all participants completed the exercise program. Therefore, all data of the LS (N = 8) and NN (N = 8) groups were used for statistical analysis. The participants performed the exercises >80% of the time during the intervention period based on the recording sheet. Table 1 shows the characteristics of the participants. The only significant difference between the groups was height.

**Table 1.** Characteristics of participants (mean ± SD)

	LS group (N = 8)	NN group (N = 8)
Age (y/o)	25.8 (±4.6)	23.0 (±1.1)
Height (cm)	177.2 (±5.2)	169.1 (±3.4)
Weight (kg)	65.5 (±7.7)	63.9 (±10.1)
External rotation muscle strength (Nm)	33.5 (±5.7)	32.0 (±4.0)
Infraspinatus CSA (cm^2^)	13.4 (±1.9)	12.5 (±1.4)

SD, standard deviation; LS, low intensity and slow movement; NN, normal intensity and normal speed; y/o, years old; CSA, cross-sectional area

### Infraspinatus CSA

Table 2 shows the infraspinatus CSA. The significant main effect was presented between factors of time (F = 7.19, p <0.01), and a significant interaction effect between groups and time was also found (F = 7.84, p <0.01). The post-hoc test indicated that the infraspinatus CSA significantly increased in the LS group between baseline and 8 weeks. In the NN group, no significant changes were found in the infraspinatus CSA. The percentage of change in the infraspinatus was significantly greater in the LS group (7.3% ± 0.1%) than in the NN group (−0.1% ± 0.0%).

**Table 2.** Infraspinatus CSA (mean ± SD)

CSA (cm^2^)	Baseline	4 weeks	8 weeks
LS	13.4 (±1.9)	13.7 (±2.2)	14.4 (±2.5)*
NN	12.5 (±1.4)	12.5 (±1.5)	12.5 (±1.3)

*: significant difference compared with baseline (p <0.05)

CSA, cross-sectional area; SD, standard deviation; LS, low intensity and slow movement; NN, normal intensity and normal speed

### Muscle strength of the external rotator

Table 3 shows the muscle strength values. No significant main or interaction effects were found in either isometric or isokinetic muscle strength.

**Table 3.** External rotation muscle strength (mean ± SD)

Isometric (Nm)	Baseline	4 weeks	8 weeks
LS	33.5 (±5.7)	34.8 (±6.5)	35.5 (±6.3)
NN	31.6 (±3.9)	35.1 (±4.1)	33.8 (±5.6)
60°/s concentric (Nm)			
LS	21.8 (±8.8)	22.8 (±6.0)	22.5 (±3.9)
NN	25.6 (±4.0)	24.6 (±2.9)	25.1 (±4.1)
120°/s concentric (Nm)			
LS	21.0 (±7.4)	21.5 (±6.3)	21.9 (±6.2)
NN	23.0 (±4.6)	23.5 (±3.1)	24.0 (±4.0)
60°/s eccentric (Nm)			
LS	28.8 (±7.6)	30.4 (±6.7)	29.9 (±8.2)
NN	30.6 (±3.9)	32.1 (±4.3)	33.6 (±6.8)
120°/s eccentric (Nm)			
LS	28.8 (±9.5)	29.4 (±8.5)	29.0 (±8.7)
NN	32.8 (±4.0)	30.4 (±4.0)	33.9 (±8.5)

There were no significant differences in any values.

SD, standard deviation; LS, low intensity and slow movement; NN, normal intensity and normal speed

### Infraspinatus EMG

Table 4 shows the EMG values of the infraspinatus. No significant differences in averaged EMG activity were found between the two groups. However, the LS group had significantly greater integrated EMG activity than the NN group.

**Table 4.** Infraspinatus EMG (mean ± SD)

	LS	NN	
Averaged EMG (% MVC)	14.5 (±4.6)	22.1 (±8.0)	p = 0.12
Integrated EMG (% MVCs)	1,243.6 (±533.2)	533.0 (±241.2)	p <0.01

EMG, electromyography; SD, standard deviation; LS, low intensity and slow movement; NN, normal intensity and normal speed; MVC, maximal voluntary isometric contraction

## Discussion

This study investigated whether an 8-week intervention of low-intensity shoulder external rotation exercises with slow movement increases the muscle strength and CSA of the infraspinatus. We hypothesized that infraspinatus CSA would significantly increase following low-intensity exercises with slow movement after 8 weeks. To our knowledge, this study is the first to report the effect of LS exercise on the CSA of infraspinatus hypertrophy.

No significant difference in the averaged infraspinatus EMG activity, measured during one repetition of shoulder external rotation exercises, was found between the two groups. Bitter et al. investigated infraspinatus EMG activity with various intensities and reported that infraspinatus activity did not increase even when the intensity was increased from 10% to 70% of shoulder isometric external rotation strength^7)^. Additionally, Cools et al. noted that high middle part of trapezius EMG activity occurred during shoulder external rotation, with the arms at the side^22)^. Therefore, in the NN group, the infraspinatus EMG activity did not increase with the increase in exercise load because of the compensation by the middle part of trapezius muscle. By contrast, the LS group had significantly greater infraspinatus integrated EMG activity, measured during one set of exercises, than the NN group. This finding indicated that the infraspinatus had a greater workload during exercise in the LS group than that in the NN group. Infraspinatus hypertrophy was observed only in the LS group because the workload of the infraspinatus contributes to muscle hypertrophy. Burd et al. investigated whether the time in which the muscle was under loaded tension during low-intensity resistance exercises affects the synthesis of muscle proteins in the quadriceps muscles of healthy male participants^23)^. They observed significant protein synthesis after slow-movement exercises. They concluded that the time the muscle was under tension during exercise was important in increasing protein synthesis and optimizing muscle growth. As known, eccentric contraction exercises have greater effects on muscle hypertrophy and muscle strength than concentric contraction exercises^24)^. However, Moore et al. reported contradictory results^25)^. They investigated whether training-induced increase in the size and strength of the elbow flexor muscles differed between muscle contraction types (eccentric or concentric) when the total external workload was the same. They found no significant differences in the increases in muscle size and strength between the two groups after 9 weeks, so they concluded that increases in muscle size and strength with short-term resistance training were unrelated to the muscle contraction type when matched for both exercise intensity and total external workload. In the present study, the mechanical stress on the infraspinatus was larger in the LS group than in the NN group because of the longer contraction times caused by slow movement, which contributed to the increase in the workload of the infraspinatus throughout the exercise. These results, taken together with those of previous studies, suggest that the larger mechanical stress on the infraspinatus caused infraspinatus hypertrophy and therefore was observed only in the LS group.

In the LS and NN groups, no significant increase in shoulder external rotation strength was found. The absence of an increase in muscle strength in both groups can be explained by the principle of resistance training, that is, “principle of specificity.” In this principle, the maximal benefit may be derived from exercise styles that closely simulate those used in the specific activities. Regarding movement speed, muscle strength increased most at the speed same as that used during training^26)^. In the present study, we evaluated isometric and isokinetic muscle strength. These evaluated values possibly did not reflect the effect of the intervention because the contraction style and movement speed differed from exercises in the actual intervention. Neural adaptation is another factor that contributes to muscle strength and hypertrophy. A previous study reported that at least 80% of maximal muscle strength is needed for neural adaptation^27)^. Moreover, a meta-analysis that investigated which load was the most effective for muscle strengthening reported that 60% of maximal muscle strength had the largest effect size in participants without training experience compared with 80% in participants with training experience^28)^. Another meta-analysis study noted that 85% of maximal strength had the largest effect size in athletes^29)^. In view of these studies, it appears necessary that training would start from at least 60% of maximal strength and gradually increase up to approximately 85% to achieve muscle strengthening. Additionally, no previous study has reported an increase in muscle strength because of low-intensity training for the infraspinatus^10^^,^^12)^, which is consistent with our results. Because the averaged infraspinatus EMG activity was approximately 20% MVC in both groups in this study, it appears that the load used was insufficient for muscle strengthening. Therefore, the evaluated muscle strength was different from the principle of specificity of training, and the intensity was too low to cause neural adaptation of the infraspinatus; thus, the effect of the exercise possibly did not influence the shoulder external rotation strength despite the increase in the CSA. Previous studies reported that low- intensity and slow-movement exercises improved muscle strength as well as the CSA and muscle thickness of lower limb muscles^13^^–^^15)^. In this study, LS condition significantly increased the CSA of the infraspinatus; however, there was no significant increase in shoulder external rotation strength. These differences may result from very light load (500 g) of the LS group. Previous studies used 50% of 1RM as the low-intensity condition. In this study, a very light load was used in the LS group in order to prevent compensation by the deltoid muscles and to examine the effects of clinically used loads. In the LS group, 500 g was equivalent to 4% of the maximum isokinetic muscle strength and a very low load compared to previous studies of the lower limb muscles. Therefore, the results of muscle strength may differ from those of previous studies.

Clinically, atrophy of the rotator cuff muscles is observed frequently in participants with shoulder disorders. To solve this problem, low-intensity exercise is generally considered more appropriate for rotator cuff muscles^20^^,^^21)^. Although a few reports have indicated that low-intensity exercises caused muscle strengthening, no studies have indicated muscle hypertrophy^10^^–^^12)^. Additionally, because these studies involved the use of a 3-kg dumbbell, elastic band, or isokinetic dynamometer, the load appears to be too high for a patient who is sensitive to excessive stress, even using these loads. In this study, we used a 500-g dumbbell, which was much lighter than that in previous studies, and we observed infraspinatus hypertrophy by adjusting the movement speed. Therefore, it may be an effective training method for developing infraspinatus hypertrophy in patients who should avoid excessive stress in the early phase of rehabilitation.

This study has some limitations. First, the study participants were healthy young men and the sample size was small; thus, it is unknown whether the training protocol used could be effective for patients with shoulder disorders. Since weakness of rotator cuff muscle strength may exist in patients with shoulder disorder, the effect of muscle strengthening may be more likely to occur. Further interventional study is needed to solve this limitation. Second, although significant differences in muscle CSA were observed, no significant differences were found in isometric and isokinetic muscle strength. Because it is possible that the angular velocity for evaluating isokinetic muscle strength in this study was too fast for the exercise conditions and muscle function also includes muscle power and endurance, further study is needed to clarify the effects on muscle strength and functions by comparing these factors.

## Conclusion

In this study, we investigated the effect of an 8-week low-intensity shoulder external rotation exercise with slow movement. Our results suggest that low-intensity exercises with slow movement significantly increased the CSA of the infraspinatus compared with normal-intensity exercises with normal speed.

## Conflict of Interest

None. No benefits in any form have been or will be received from a commercial party related directly or indirectly to the subject of this article.
